# Diagnostic accuracy of positron emission tomography/computed tomography-driven biopsy for the diagnosis of lymphoma

**DOI:** 10.1007/s00259-020-04913-9

**Published:** 2020-06-15

**Authors:** Alessandro Broccoli, Cristina Nanni, Alberta Cappelli, Francesco Bacci, Alessandro Gasbarrini, Elena Tabacchi, Carlo Piovani, Lisa Argnani, Riccardo Ghermandi, Elena Sabattini, Rita Golfieri, Stefano Fanti, Pier Luigi Zinzani

**Affiliations:** 1 Institute of Hematology “L. e A. Seràgnoli”, University of Bologna, via Massarenti 9, 40138 Bologna, Italy; 2 Nuclear Medicine, Medicina Nucleare Metropolitana, Sant’Orsola-Malpighi Hospital, University of Bologna, via Massarenti 9, 40138 Bologna, Italy; 3 Radiology Unit, Sant’Orsola-Malpighi Hospital, via Massarenti 9, 40138 Bologna, Italy; 4 Haematopathology Unit, Sant’Orsola-Malpighi Hospital, via Massarenti 9, 40138 Bologna, Italy; 5 Oncological and Degenerative Spine Surgery, Institute of Orthopaedics “Rizzoli”, via Pupilli 1, 40136 Bologna, Italy

**Keywords:** Diagnostic accuracy, Positron emission tomography, Computed tomography, Driven biopsy, Diagnosis, Lymphoma

## Abstract

**Introduction:**

Biopsy of affected tissue is required for lymphoma diagnosis and to plan treatment. Open incisional biopsy is traditionally the method of choice. Nevertheless, it requires hospitalization, availability of an operating room, and sometimes general anesthesia, and it is associated with several drawbacks. Fluorodeoxyglucose positron emission tomography/computed tomography (PET/CT) can be potentially used to drive biopsy to the most metabolically active area within a lymph node or extranodal masses.

**Methods:**

A study of diagnostic accuracy was conducted to assess the performance of a PET-driven needle biopsy in patients with suspect active lymphoma.

**Results:**

Overall, 99 procedures have been performed: three (3.0%) were interrupted because of pain but were successfully repeated in two cases. Median SUVmax of target lesions was 10.7. In 84/96 cases, the tissue was considered adequate to formulate a diagnosis (diagnostic yield of 87.5%) and to guide the following clinical decision. The target specimen was a lymph node in 60 cases and an extranodal site in 36. No serious adverse events occurred. The sensitivity of this procedure was 96%, with a specificity of 100%, a positive predictive value of 100%, and a negative predictive value of 75%.

**Conclusion:**

Patients can benefit from a minimally invasive procedure which allows a timely and accurate diagnosis of lymphoma at onset or relapse.

## Introduction

The biopsy of an affected organ or tissue is highly desirable in establishing the diagnosis of cancer, and it is absolutely mandatory to confirm an initial suspect of a lymphoproliferative disease, both at onset and in case of suspected relapse. Adequate amounts of tissue taken from the most representative nodal or extranodal lesion permit a correct classification of the disease within well-recognized lymphoma subcategories through the application of the required immunohistochemical analyses and of molecular biology or cytogenetic techniques both on paraffin embedded and on fresh tissue [1].

Open incisional biopsy (OIB) is traditionally regarded as the method of choice for diagnosing tumors and tumor-like lesions [2]. It usually requires hospitalization, the availability of an operating room, and often general anesthesia. While OIB is still considered the gold standard because of its accuracy (approximating 100%), it is seldom associated with drawbacks, which include morbidity and possible surgical complications, and it is a more time- and resource-consuming approach. The development of imaging-guided core needle biopsies has partly overcome these disadvantages [3, 4]: computed tomography (CT), ultrasound (US), and fluoroscopy-guided procedures are in fact easy to perform, safe, less invasive than OIB, and cost-effective. This is particularly true when deep abdominal or thoracic lesions, as well as sites such as the skeleton or the spine, are concerned. For this reasons, imaging-guided techniques nowadays play an ever-growing role in the diagnosis of active disease in lymphoma patients, in whom any nodal district or potentially any extranodal site can be affected by the neoplastic tissue [5–10].

The diagnostic rate of CT-guided or US-guided core needle biopsy is usually acceptable, as these procedures permit the collection of amounts of tissue adequate to accomplish a specific diagnosis in a significant proportion of cases [3, 5, 7–11]. Furthermore, the repeated biopsy rate and the incidence of complications are very low. No significant risk in terms of local recurrence along the core needle biopsy tract has been reported.

Despite these advantages, the accuracy of CT- and US-guided core needle biopsies for a specific diagnosis of lymphoma approximates 85–90% for nodal localizations, and it is not clearly established—although markedly reduced—for extranodal sites [3, 5, 7–11]. In most extranodal areas, in fact, and with particular regard to the skeleton [12], lymphoma-related morphological changes are hard to be recognized on CT images only, whereas they appear more clearly if a functional imaging technique such as ^18^F-fluorodeoxyglucose (FDG) positron emission tomography (PET) is applied [13, 14]. FDG-PET is able to highlight areas of active lymphoma both in lymph nodes and other tissues (including the bone and most of the parenchymas) with a very high sensitivity in comparison with conventional imaging, given the documented—although variable—avidity of the lymphoma tissue for FDG [15]. Importantly, PET can provide pathological results even before lymphoma-related radiological changes occur (e.g., in bones) and can accurately discriminate between residual fibrotic tissue after therapy and disease persistence or relapse. Thus, the possible application of FDG-PET, when combined with CT scan (PET/CT), to drive the biopsy toward the most metabolically active lesion, to the most active area within a bulky mass or directly into a bone or parenchymal focal localization is theoretically of great advantage.

Here, we present the results of a study in which PET and CT fused images were used as a guide to drive the core needle biopsy directly into the most suitable target in case of suspected lymphoproliferative disorder at onset or at relapse.

## Methods

### Study design and overall conduct

This was an exploratory interventional monocentric study on patients with documented FDG-avid PET-positive findings suspicious for an active lymphoproliferative disease, either at first documentation or in case of suspected relapse in patients with previous known lymphoid malignancy. A multidisciplinary team (hematologists, nuclear medicine physicians, interventional radiologists, orthopedic surgeons, and hematopathologists) was involved. Hematologists were responsible for the initial clinical evaluation of patients and formally gave the indication for the biopsy. They also followed patients after the procedure, discussing the diagnosis and deciding regarding any subsequent treatment (in case of a biopsy conclusive for a hematological or oncological disease, i.e., true positive). Patients with a non-diagnostic biopsy or with inconclusive findings were followed for at least 1 year in order to discriminate between false negative and true negative results.

Patients aged 18 years or older were considered eligible provided they showed FDG-avid findings suspicious for lymphoma or any lymphoid disease requiring histological confirmation. Patients were excluded if they displayed palpable superficial adenopathies which could be surgically excised (unless proved metabolically negative) and in case a core needle biopsy was judged risky or contraindicated (active bleeding, hemorrhagic diathesis, anatomical impediments).

The study protocol was approved by the local ethic committee, in accordance with the Italian law and in compliance with the declaration of Helsinki. Patients provided a written informed consent. Patients were consecutively involved to avoid selection bias.

### Imaging and biopsy procedures

**Fig. 1:**
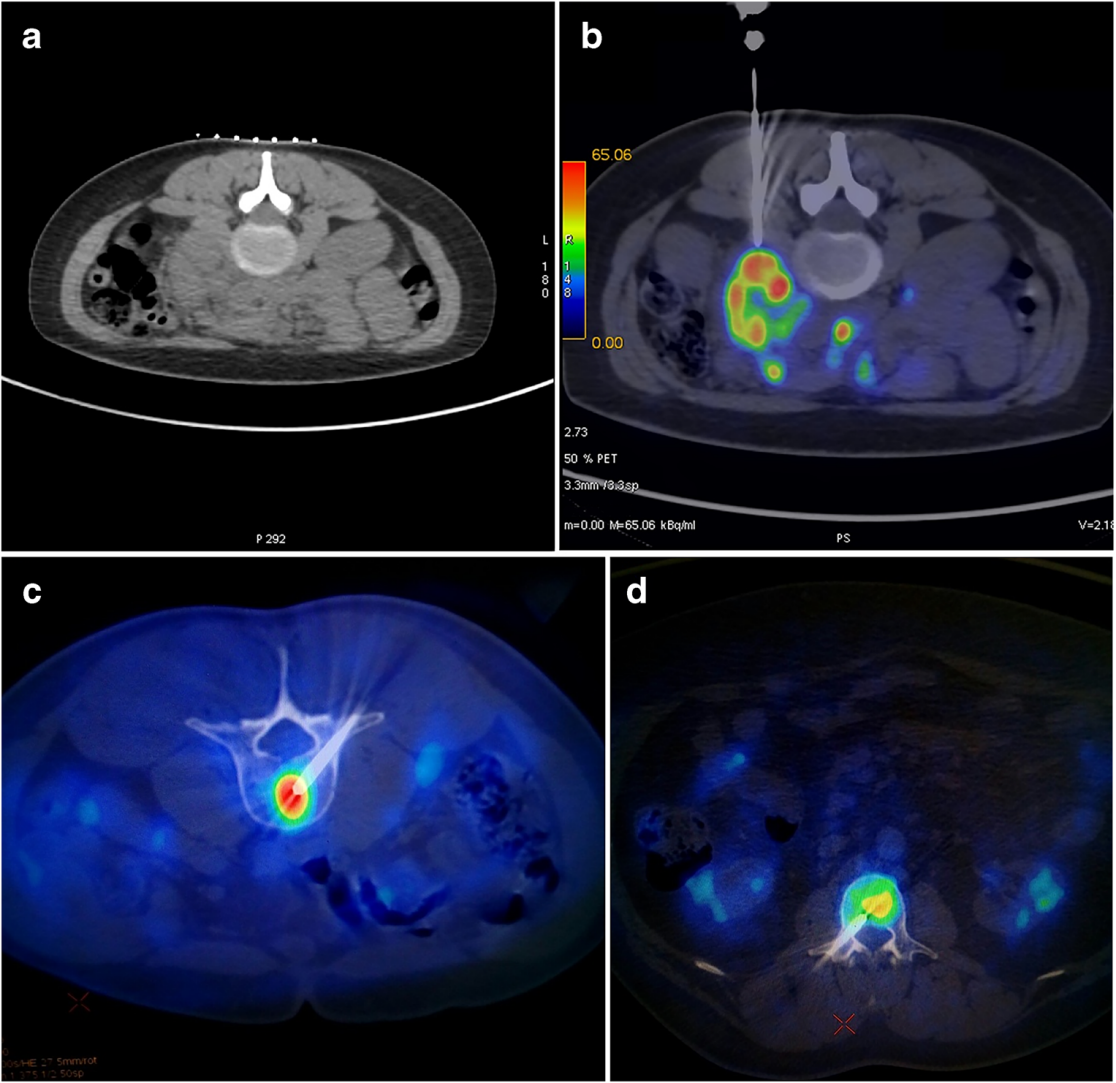
Left lumboaortic bulky adenopathy (**a**) with heterogeneous FDG-PET uptake within the mass (**b**): PET/CT fused images help the interventional radiologist reach the most metabolically active portion of the tumor (SUVmax 36.6) for adequate sampling. Final diagnosis was consistent with double expressor (MYC/BCL2-positive) diffuse large B cell lymphoma, with no *MYC* rearrangements. Focal FDG-PET hypermetabolism within the body of the third lumbar vertebra (**c** and **d**) in two patients with a history of diffuse large B cell lymphoma during follow-up. SUVmax of the target lesions were 9.5 (**c**) and 7.9 (**d**), respectively. Histological analysis of the specimens confirmed relapse in both cases

The fusion of PET and CT images was required before starting the biopsy procedure in order to individuate the most suitable target and to plan the shortest and safest trajectory of the needle. PET images were acquired on a 3D tomograph for 2 min/bed position, including the whole body. Concomitant low-dose CT or contrast-enhanced CT scans were performed, both for attenuation correction and as an anatomical map (in-line method). Target lesions were chosen among the most metabolically active according to maximal standardized uptake value (SUVmax) and according to their accessibility; if the uptake was heterogeneous within a bulky nodal lesion, a parenchyma, or a bone (Fig. 1), the area with the highest SUVmax was considered the most adequate for tissue sampling, thus trying to avoid areas of necrotic or scar tissue. PET images acquired within 30 days before the day of the biopsy could be considered suitable to drive the procedure, as well, provided they could be fused with CT images (either with or without contrast enhancement) acquired during the biopsy itself (off-line method).

Once the interventional radiologist (or the orthopedic surgeon, in case of skeletal or vertebral biopsy) and the nuclear medicine physician had selected the target and had chosen the appropriate window of access, local anesthesia with 1% lidocaine was administered and a coaxial 12–18-gauge needle was positioned within the lesion. Repeated PET/CT scans were used to monitor the needle progression into the most active area of the lesion and to perform the biopsy.

The bioptic sample was preliminary evaluated on-site by a dedicated hematopathologist by means of touch preparations or smear, trying to ensure that cellulated areas were caught and asking for additional sampling if necrotic or acellulated tissue was biopsied. The final pathology report was delivered within a week following the international standards [1].

### Study objectives and end points

The primary objective of the study was to determine the accuracy of a combined PET- and CT-driven biopsy (with the use of fused images) in establishing the diagnosis of lymphoma or lymphoproliferative disease at onset or at relapse. This was calculated as the ratio between all the diagnostic samples and the total of completed procedures, i.e., those ending up with a final ready-to-use diagnosis (diagnostic yield) and by establishing sensitivity and specificity of the procedure. Secondary objectives were represented by evaluation of specimen adequacy (in terms of length of the specimen, disease infiltration within the specimen, amount of bony, or scar tissue) and reduction of failures (non-diagnostic findings in terms of insufficient or out-of-target sampling) in comparison with already published results. Any adverse event (AE) occurred during treatment was encoded according to the NCI Common Terminology Criteria for AEs v. 4.03.

### Statistical analysis

This study was planned as a hypothesis-generating trial, which generates data for further studies which will formally compare the diagnostic accuracy of different methods. Given the exploratory fashion of the study, there was not a formal sample size determination. Basing on our experience, we planned to enroll 100 patients in 3 years.

Demographics and patients’ characteristics were summarized by descriptive statistics. Diagnostic accuracy was calculated through the sensitivity and specificity of the test. The confidence interval (CI) was calculated with the continuity-corrected Newcombe’s method. No formal comparison with reference standard was made, as none of the patients received a surgical OIB.

## Results

One hundred patients were considered eligible for a PET-/CT-driven biopsy between March 2016 and December 2018. Among those patients, 41 showed FDG-avid PET-positive lesions compatible with lymphoproliferative disorder at onset, whereas in 59 cases, there was a suspect of disease relapse. Patients’ characteristics are listed in Table 1. In one case, the baseline PET scan turned out to be negative, thus precluding any further evaluation within the protocol. In two patients, a core needle biopsy could not be performed due to anatomic reasons: in one case, the ureter was too close to the target lesion and could have been damaged during the procedure; in the latter case, the target abdominal mass rapidly reduced between the time of the first PET scan and the biopsy, in this sense making the suspect of lymphoma extremely unlikely.

**Table 1:** Characteristics of enrolled patients and procedure details

Enrolled patients (undergoing at least one procedure), *N*	97
Median age, years (range)	63 (22–92)
Sex (male/female), *N*	57: 43
Inpatient/outpatient, *N*	32: 68
Pre-biopsy setting, *N*	
Suspected lymphoma at onset	41
Suspected lymphoma at relapse	59
Total procedures, *N*	99
Interrupted because of an adverse event (no sampling)	3
Repeated procedures among those interrupted	2
Biopsy site (among all the 96 completed procedures), *N*	
Lymph node	60
Extranodal site (bone 22; soft tissue 7; liver 5; kidney 1; adrenal 1)	36
Median SUVmax of target lesion (range)	10.7 (1.6–67.9)

**Fig. 2:**
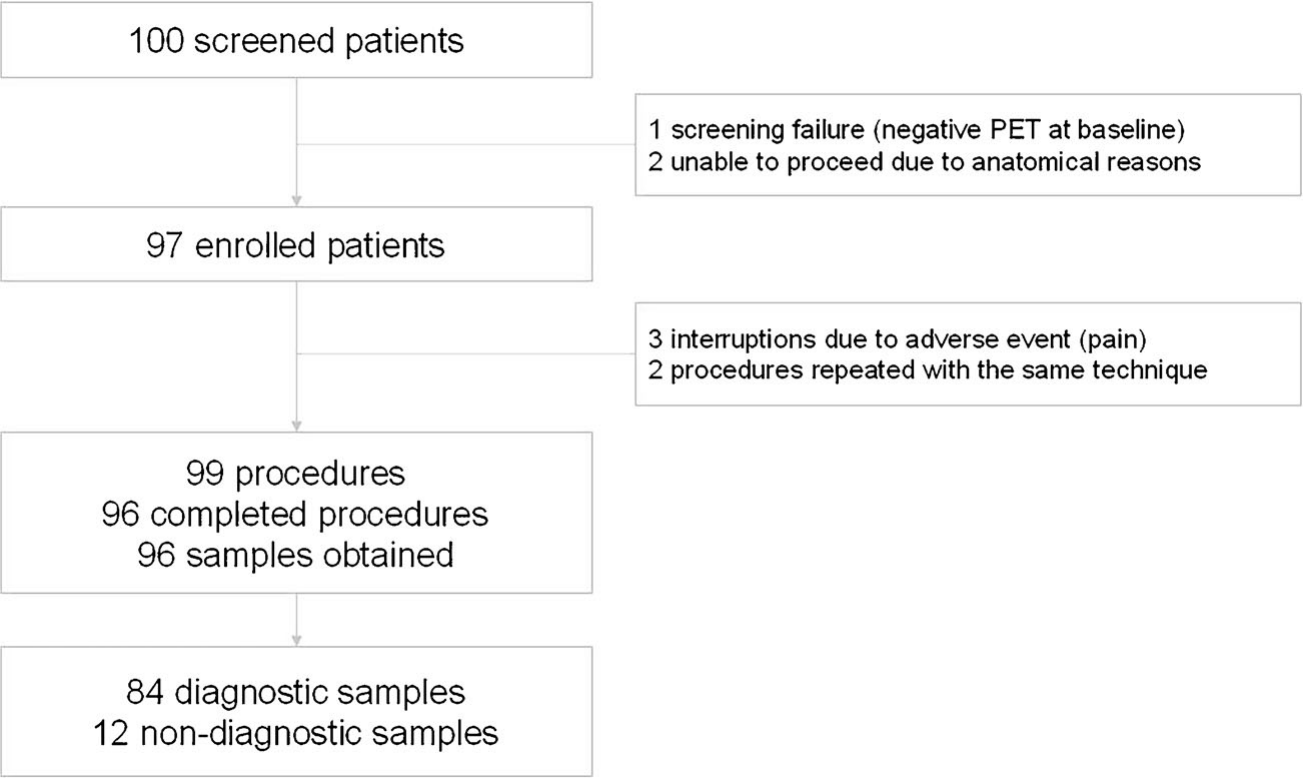
Study layout

For these reasons, 97 patients underwent at least one procedure. In three instances, an initiated procedure needed to be interrupted because of an adverse event (see next paragraphs for details), but in two cases, the procedure could be repeated with the same technique, within a few days and without complications; therefore, 99 procedures have been performed overall (Fig. 2). Taking into account all the 96 completed procedures, that means all of those ending with tissue sampling, a lymph node was the target of the biopsy in 62.5% of the cases, and an extranodal site was chosen as the most relevant site in the remaining 37.5% of the cases. Importantly, the skeleton was the most prevalent extranodal site (22 cases, 22.9% of cases), followed by soft tissue (7 cases), liver (5 cases), kidney, and adrenals (one case each).

### Diagnostic yield and reliability of the procedure

Taking into account the 96 processed samples, 84 were considered adequate to accomplish a reliable final diagnosis, with a diagnostic yield of 87.5% (Table 2). More precisely, the initial suspect of active lymphoma was confirmed in 62 cases, along with one case of chronic lymphocytic leukemia and one case of acute lymphoblastic leukemia: in this latter case, the diagnosis was obtained by the biopsy of a PET-positive area within the bone marrow, in absence of any other nodal or extranodal positive finding. In particular, the following lymphoma histologies could be clearly assessed: follicular lymphoma (19 cases), diffuse large B cell lymphoma (16), Hodgkin lymphoma (11), marginal zone lymphoma (6), indolent lymphoma not otherwise specified (4), anaplastic large-cell lymphoma (2), lymphoplasmacytic lymphoma, T cell lymphoma (not otherwise specified), mantle cell lymphoma, and plasmablastic lymphoma (one case each). In eight cases, a diagnosis of solid tumor or lymph node metastasis by epithelial neoplasm was made (all adenocarcinoma).

**Table 2:** Study outcomes and diagnostic reliability

Failure rate (interrupted procedure), *N* (%)	3/99 (3.0%)
Chance to repeat any failed procedure (*), *N* (%)	2/3 (66.7%)
Rate of non-diagnostic sampling (**), *N* (%)	12/96 (12.5%)
Diagnostic yield (**), *N* (%)	84/96 (87.5%)
Diagnostic samples, *N* Lymphoma Follicular lymphoma Diffuse large B cell lymphoma Hodgkin lymphoma Marginal zone lymphoma Indolent lymphoma (not otherwise specified) Anaplastic large-cell lymphoma Lymphoplasmacytic lymphoma Mantle cell lymphoma Peripheral T cell lymphoma (not otherwise specified) Plasmablastic lymphoma Chronic lymphocytic leukemia Acute lymphoblastic leukemia Other (non-hematological/non-oncological findings) Solid tumor or metastasis	84 62 19 16 11 6 4 2 1 1 1 1 1 1 12 8
Sample adequacy for clinical decisions (***), *N* (%)	84/84 (100%)

(*) Two patients repeated a procedure previously interrupted: this explains the number of 99 procedures performed in 97 enrolled patients. (**) Calculated on all completed procedures, i.e., those ending up with adequate tissue sampling. (***) Accounts for only diagnostic samples

In 12 cases, the obtained tissue was adequate to exclude any hematological or oncological findings, indicating no evidence of neoplasm. All these patients were followed up for a median period of 6 months (range, 1–25 months): three of them underwent a new biopsy (with a different technique), confirming a diagnosis of lymphoma (false negative cases according to the test). In all the other instances, no evidence of malignancy was documented during follow-up (true negative cases). Taking into account all the diagnostic specimens, the sensitivity of the test is 96% (95% CI 0.886–0.989), and the specificity is 100% (0.946–1.000). Consequently, the positive predictive value of the test is 100% (95% CI 0.946–1.000), with a negative predictive value of 75% (95% CI 0.641–0.835). The latter rises to 85% (95% CI 0.752–0.915) when taking into account the probability of being free of lymphoma (instead of a neoplastic disease in general) when the biopsy shows no lymphoma involvement.

Twelve samples (12.5%) were non-diagnostic for either insufficiency or inadequacy of the tissue or because it was highly unrepresentative. Patients with a non-diagnostic test were followed up for a median period of 8.5 months (range, 1–14 months): four of them received a new biopsy, obtaining a diagnosis of lymphoma in three cases and a diagnosis of bladder carcinoma in one case. Eight patients are still in clinical follow-up, without any evidence of disease.

**Fig. 3:**
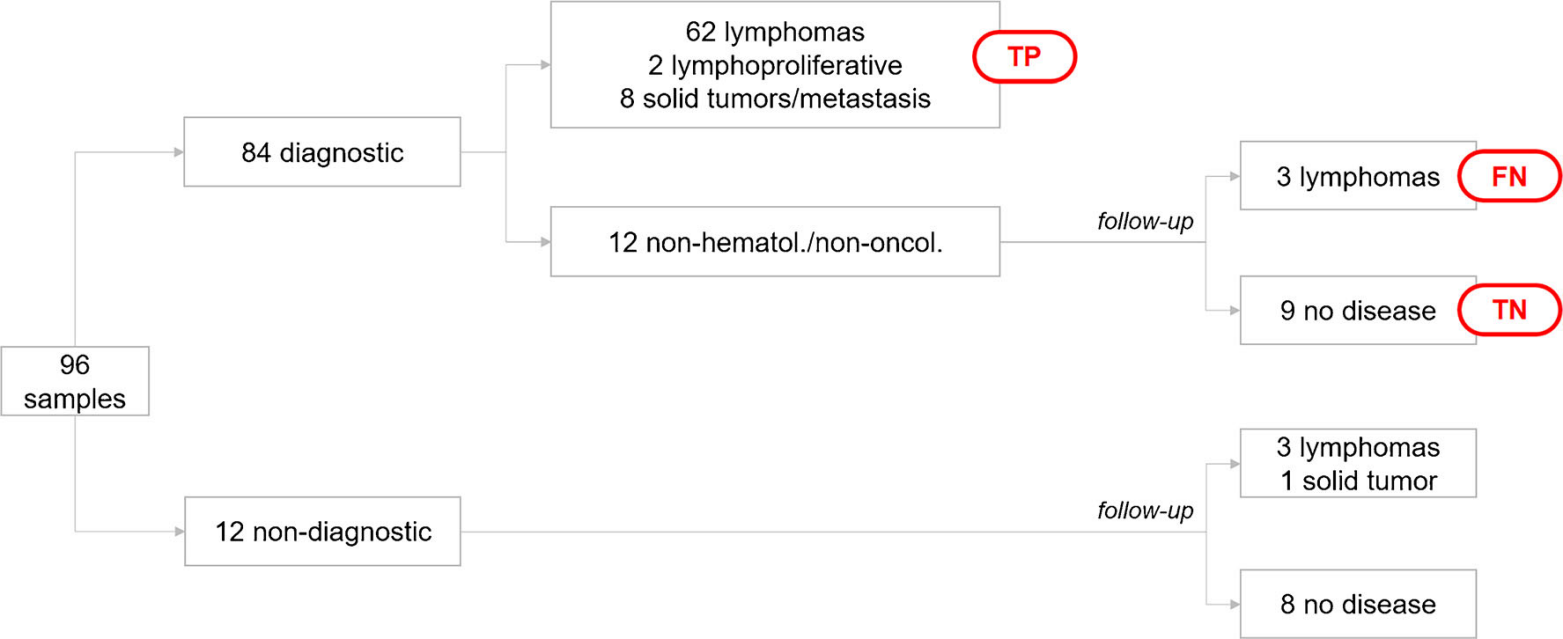
Flow chart of results. TP, true positive; FN, false negative; TN, true negative. All patients with a non-diagnostic finding or a non-hematological/non-oncological finding were followed-up according to protocol procedures (see text for explanation)

Figure 3 outlines the diagnostic flow and includes the results after patients follow-up.

### SUVmax analysis

The median SUVmax at the sites chosen as the target of the biopsy was 10.7, ranging between 1.6 and 67.9. Among the patients with a final diagnosis of lymphoma (or lymphoproliferative disorder), the median SUVmax was 11.5 (3.0–67.9), and among those with a diagnosis of solid neoplasm, it was 10.0 (4.0–31.0). Patients with non-hematological/non-oncological findings displayed a lower median SUVmax (6.6, ranging between 1.6 and 15.9).

### Specimen characteristics

The mean length of collected specimens was 10 mm (assumed with an absolute error of ± 1 mm), ranging from 3 to 30 mm, and with a standard deviation (SD) ± 6 mm. The mean amount of pathologic infiltrate in collected samples was 70%, ranging from 0 to 100%, with a SD of ± 34%. Samples with 0% pathologic infiltrate were judged as non-diagnostic or negative for disease, depending on the amount and type of the overall tissue. The mean proportion of fibrosis (scar tissue) or bone was 30%, ranging from 0 to 100% (SD ± 30%). Samples containing a large amount of fibrous tissue or bone (≥ 75%) or lacking a significant amount of pathologic infiltrate (≤ 20%) were judged as non-diagnostic or negative for disease.

### Extranodal sites

Among the 36 patients who were biopsied at an extranodal site, 30 specimens were adequate to accomplish a correct diagnosis, with a diagnostic yield of 83.3%. More specifically, a diagnosis of lymphoma was possible in 19 cases (52.8%) and of acute lymphoblastic leukemia in one case; in three instances, histological findings were consistent with a diagnosis of metastatic adenocarcinoma. Unaffected tissue was found in seven cases. Two of the patients with inconclusive findings received a diagnosis of lymphoma after respectively an intestinal biopsy and a bone biopsy performed outside of this trial. The median SUVmax of the target lesion in patients affected by extranodal lymphoma was comparable with what was observed in the whole lymphoma patient population (11.5, ranging between 4.5 and 36.7).

### Adverse events

AEs were rare and always mild and transient. Neither grade three-four nor serious AE was reported (Table 3). All patients who underwent a procedure on an outpatient basis never required hospitalization due to an AE. Pain (grade 2) during the procedure was recorded in four cases out of 99 procedures (4.0%) and was responsible for the interruption in 3 cases. In all cases, complete pain relief was achieved upon interruption of the procedure, and no drug intervention was required. Importantly, the same technique could be applied again to accomplish the biopsy in 2/3 interruptions (66.7%), without any inconvenience. Hematoma within the biopsy site (grade 1) was documented in three cases (3.0%). This was painless, always asymptomatic, and required no intervention apart from patient monitoring with echosonography for at least 2 h after the procedure. In none of the cases, the hematoma increased in size. Contrast medium extravasation was observed in one case (1.0%), as well as contrast medium-associated cutaneous rash (1.0%). The latter complication was extremely mild and transient (grade 1) and did not require either delay in the procedure or drug intervention.

**Table 3:** Summary of adverse events

Pain, *N* Requiring interruption of the procedure Resolving upon interruption of the procedure Requiring any drug intervention	4 3 4 0
Hematoma within the biopsy site, *N*	3
Contrast medium extravasation, *N*	1
Contrast medium-associated cutaneous rash, *N* Requiring interruption of the procedure Resolving upon interruption of the procedure Requiring any drug intervention	1 0 0 0

## Discussion

Safe and timely biopsy is a prerequisite for optimal lymphoma and cancer care in general, and a moderately invasive, sensitive, and specific method for image-guided biopsy is highly desirable. FDG-PET currently provides the best tool for diagnostics and has shown huge potential for guiding therapy in lymphomas: an optimized minimally invasive procedure that allows an accurate diagnosis—in particular when standard open lymph node excision is not feasible—could represent a novel standard technique.

Data reported in literature using a PET/CT-driven biopsy approach are increasing progressively but mainly gather patients affected by various malignancies and inflammatory diseases; therefore, an unequivocal conclusion regarding the role of a PET/CT-driven bioptic procedure in a specific neoplastic setting is hard to be drawn [13, 16–22]. Importantly, all these published series include a relatively small percentage of patients affected by lymphomas and lymphoproliferative disorders, which rarely exceeds 20% of the enrolled patients. Nevertheless, authors agree that the combination of PET and CT information in aiding needle placement is of great benefit regardless the site of the biopsy, especially in those cases with a poor radiological correlation: this is confirmed by the high diagnostic accuracy rates reported, all above 90% [19–21].

Here, we have presented the largest prospective clinical trial specifically involving patients with suspected lymphoma (or lymphoproliferative disorders in general) at onset or at relapse in which a needle biopsy of the affected anatomical structure was driven by fused CT and PET images. Importantly, this study has demonstrated a high diagnostic yield, with 87.5% of specimens being diagnostic, with an accuracy of 96%. This indicates that a PET/CT-driven procedure is reliable in obtaining a ready-to-use histopathological diagnosis, as it helps to select the most clinically meaningful lesion (or the most active area within a mass) in terms of FDG hypermetabolism, thus being adequate to confirm the clinical suspect of an active lymphoproliferative neoplasm. In addition, the rapid on-site evaluation of the specimen we have applied throughout the study further optimizes the performance of the approach by assessing the adequacy of the tissue, thus reducing the incidence of off-target sampling and biopsy repetition. Findings negative for malignancy were described in 14.3% of all the conclusive cases, and a diagnosis of hematological malignancy was overall excluded in 23.8% of all the diagnostic specimens. The negative predictive value was 75% (probability of being free from any neoplastic disease when the biopsy was negative for malignancy), which rose to 85% when referring to the probability of being free of lymphoma instead of any neoplasm in general. For this reason, patients with negative pathological findings, albeit displaying PET findings suspicious of malignancy, require a stringent follow-up and, ultimately, new tissue sampling if diagnostic doubts persist.

PET guidance putatively increases the yield of tissue representative of hematological malignancy, although PET-positive sites may also reflect inflammatory or reactive states or may be indicative of malignancies other than hematological. As far as patients with a clinical suspect of lymphoma are concerned, CT- and ultrasound-guided biopsies have a relatively lower capacity of sampling nodal or extranodal tissue clearly representative of lymphoma. This can be stated by taking into account the few prospective series described in literature [9, 14, 23–25], though data are generally hard to be compared: some studies are retrospective and only report outcomes in patients with a known diagnosis of lymphoma, thus with the limitation of a selection bias [8, 26]; other trials are only limited to superficial nodal lesions, in this sense excluding both deep nodes and extranodal sites which are the major indication for a needle biopsy approach [6, 7, 11].

The low rate of complications described in our experience makes this method extremely safe and easily affordable on an outpatient basis and also in patients with comorbidities, for whom general anesthesia and invasive surgery can be definitely contraindicated. These data favorably compare with previous experiences reported in literature with oncological patients [13, 14, 16, 17, 20–22].

## Conclusion

PET/CT-guided biopsy is an effective, safe, minimally invasive procedure which allows a histological diagnosis of lymphoma in most cases at onset or relapse in patients with deep nodal or extranodal lesions. It has a relevant translational potential to hematology, oncology, and to any other field in which sampling of FDG-avid lesions represents a significant step to improve patient management and to tailor treatment strategies.

## Data Availability

Original data are available on request.
